# MRI Features Predictive of Aicardi-Goutieres Syndrome

**DOI:** 10.15844/pedneurbriefs-29-1-7

**Published:** 2015-01

**Authors:** J. Gordon Millichap

**Affiliations:** 1 Division of Neurology, Ann & Robert H. Lurie Children's Hospital of Chicago, Chicago, IL; 2 Departments of Pediatrics and Neurology, Northwestern University Feinberg School of Medicine, Chicago, IL

**Keywords:** Pediatric neuroradiology, Leukodystrophies, Aicardi-Goutières syndrome

## Abstract

Investigators from Children's National Health System Washington, DC, USA: Harvard University, Boston, USA; Leeds Teaching Hospitals, UK; and other international centers review a series of patients with MRIs selected from IRB-approved leukodystrophy biorepositories to identify MRI patterns for recognition of early-onset Aicardi-Goutieres (A-G) syndrome and scored for a panel of radiologic predictors.

Investigators from Children's National Health System Washington, DC, USA: Harvard University, Boston, USA; Leeds Teaching Hospitals, UK; and other international centers review a series of patients with MRIs selected from IRB-approved leukodystrophy biorepositories to identify MRI patterns for recognition of early-onset Aicardi-Goutieres (A-G) syndrome and scored for a panel of radiologic predictors. MRI features for pattern recognition of A-G syndrome are temporal lobe swelling follow-ed by atrophy with temporal horn dilatation, early global cerebral atrophy and visible calcifications (94.44% cases of A-G syndrome correctly identified with a sensitivity of 90.9% and specificity of 96.9%). The panel of MRI features predictive of A-G syndrome in young patients (mean age 1.2 years) differentiates it from other leukodystrophies such as Alexander disease, cytomegalovirus or rubella, Fukuyama congenital muscular dystrophy, and Walker-Warburg syndrome. An algorithm of early infantile leukodystrophies with and without temporal lobe swelling or temporal lobe dilation and cysts shows the differentiation of A-G syndrome from other leukodystrophies based on MRI analyses. These diseases have slightly different typical ages of onset versus A-G, and therefore slightly different ages of available MRI studies. A predominance of patients with A-G syndrome in this study had mutations in TREX1. [1]

COMMENTARY. Aicardi-Goutieres (A-G) syndrome [2] is an inherited leukoencephalopathy caused by mutations in one of five genes, including TREX1 and SAMHD1, and resulting in a phenotype of CSF chronic lymphocytosis, increased CSF alpha interferon, and a calcifying microangiopathy with abnormal CNS white matter. Autoimmune disorders such as chilblains (pernio) are associated. Classically, the diagnosis of A-G syndrome is suspected when calcifications are identified by CT scan, but with increased availability and high specificity of the MRI, the CT is no longer essential. The disease is rapidly fatal or progresses to a vegetative state. A-G syndrome is both genetically and phenotypically heterogeneous, with a range of severity from life-threatening perinatal illness to mild late infancy onset [3]. An atypical patient is reported with late-onset A-G syndrome, characterized by spastic paraparesis and a leukoencephalopathy that markedly improved during follow-up and showed resolution of the white matter abnormalities [4].

A-G syndrome is distinguished from Aicardi syndrome. Aicardi syndrome is characterized by agenesis of the corpus callosum, infantile spasms, and chorioretinal lacunae [5]. Aicardi syndrome affects mainly girls, and is rare and usually fatal in XXY males [6].
